# Airborne environmental DNA metabarcoding detects more diversity, with less sampling effort, than a traditional plant community survey

**DOI:** 10.1186/s12862-021-01947-x

**Published:** 2021-12-06

**Authors:** Mark D. Johnson, Mohamed Fokar, Robert D. Cox, Matthew A. Barnes

**Affiliations:** 1 Department of Natural Resources Management, Texas Tech University, Lubbock, TX 79409 USA; 2 Center for Biotechnology & Genomics, Texas Tech University, Lubbock, TX 79409 USA

**Keywords:** eDNA, Methods comparison, Plant genetics

## Abstract

**Background:**

Airborne environmental DNA (eDNA) research is an emerging field that focuses on the detection of species from their genetic remnants in the air. The majority of studies into airborne eDNA of plants has until now either focused on single species detection, specifically only pollen, or human health impacts, with no previous studies surveying an entire plant community through metabarcoding. We therefore conducted an airborne eDNA metabarcoding survey and compared the results to a traditional plant community survey.

**Results:**

Over the course of a year, we conducted two traditional transect-based visual plant surveys alongside an airborne eDNA sampling campaign on a short-grass rangeland. We found that airborne eDNA detected more species than the traditional surveying method, although the types of species detected varied based on the method used. Airborne eDNA detected more grasses and forbs with less showy flowers, while the traditional method detected fewer grasses but also detected rarer forbs with large showy flowers. Additionally, we found the airborne eDNA metabarcoding survey required less sampling effort in terms of the time needed to conduct a survey and was able to detect more invasive species than the traditional method.

**Conclusions:**

Overall, we have demonstrated that airborne eDNA can act as a sensitive and efficient plant community surveying method. Airborne eDNA surveillance has the potential to revolutionize the way plant communities are monitored in general, track changes in plant communities due to climate change and disturbances, and assist with the monitoring of invasive and endangered species.

**Supplementary Information:**

The online version contains supplementary material available at 10.1186/s12862-021-01947-x.

## Background

Accurate characterization of plant communities informs effective conservation, management, and restoration of communities and ecosystems [1]. Scientists and managers have historically used field-based quadrats and transect methods to visually survey plant communities [2, 3]. With methods ranging from line-point intercept and belt transects to surveys as simple as visual identification, these traditional approaches can help to determine what plant species exist on a landscape [3]. However, the quality of the results from traditional surveys rely heavily on how much resources (time and effort) are partitioned to the project [4]. Additionally, traditional surveying relies heavily on an expert’s ability to correctly identify plants, damages the plant life being surveyed, disrupts local animal populations, and can be labor-intensive and time consuming [3]. By requiring someone to determine the identity of a species, these surveys can introduce inter-observer (different results from multiple observers) and intra-observer (same observer with errors over time) errors [5].

Genetic monitoring technology such as environmental DNA (eDNA) analysis can address these limitations, providing scientists and mangers with a powerful tool for species detection. In this context, eDNA refers to the genetic material that is shed from an organism into its environment [6, 7], and researchers can analyze bulk environmental samples such as water, soil, or air to determine whether that sample contains genetic material from a species of interest, which can provide clues about species proximity in space and time. Collecting bulk samples requires no taxonomic expertise and is generally faster, cheaper, and less disruptive than traditional surveying methods [8]. Thus, eDNA methods are faster, less disruptive to the environment, and require less labor, which helps to directly address the limitations of traditional surveying methods [9, 10]. Additionally, multiple studies have found that eDNA methods have a higher sensitivity than traditional methods. For example, Jerde et al. [11] found that aquatic eDNA methods detected Silver Carp (*Hypophthalmichthys molitrix*) and Bighead Carp (*H. nobilis*) in the Chicago Area Waterway System with greater sensitivity than nets and electrofishing. Dejean et al. [12] also demonstrated that eDNA methods were more effective at detecting the invasive American bullfrog (*Lithobates catesbeianus*) in French wetlands compared to traditional auditory and visual surveys. Smart et al. [13] compared eDNA methods to traditional trapping techniques for the detection of an invasive smooth newt (*Lissotriton vulgaris vulgaris*) and found that eDNA detection probabilities were significantly higher than traditional trapping. Together, these and other studies emphasize the potential benefits eDNA analysis can provide to researchers and managers alike.

Historically, eDNA detection has been primarily applied to water [6, 14, 15] and soil samples [16–18]. The majority of airborne sampling has focused primarily on the detection of wind-borne grass pollen and its relation to human health [19–21]. However, recent work from Johnson et al. [22, 23] demonstrated that airborne eDNA can detect both anemophilous (wind pollinated) and non-anemophilous (insect pollinated) plant species. Johnson et al. [22] also found that species could be detected during a season when target species are not flowering and pollination is not occurring. Furthermore, Johnson et al. [24] found that airborne eDNA trends correspond to seasonal patterns and acute disturbances on a short-grass rangeland landscape. More recently, Aalismail et al. [25] found that airborne eukaryotic communities (including plant species) could be detected with samples from the global dust belt over the Red Sea. This indicates that airborne eDNA could be used in a similar manner to that of aquatic and sediment systems including community surveys, endangered species detection, and invasive species prevention. However, the previous studies have never actually used airborne eDNA metabarcoding to perform a whole community survey on a specific plant community. These studies have focused on either human health impacts of dust, specifically pollen, or single species identification. Therefore, we hypothesize that eDNA metabarcoding could be applied to survey entire plant communities.

Metabarcoding describes the process of using next-generation sequencing technologies to analyze various samples (e.g., eDNA, gut contents, microbiomes, tissue, etc.) and assess the total biodiversity of that sample, rather than focus on a single-species [8]. Next-generation sequencing technology is a massively parallel approach that allows researchers to quickly sequence thousands of sequences at once [26]. As metabarcoding technology continues to improve and costs decrease, the field of eDNA research is shifting from single-species approaches toward using metabarcoding for a more comprehensive and efficient study of whole communities [8]. Studies in aquatic systems have shown that metabarcoding can equal or exceed the performance of traditional field based methods. For example, in aquatic systems, Valentini et al. [27] found that eDNA metabarcoding could more accurately detect both bony fish and amphibians compared to auditory, visual, and collection-based methods. Recently, McClenaghen et al. [28] used eDNA metabarcoding to detect deep sea fish and found that the eDNA method gave similar results to conventional field surveys while having a much lower sampling effort requirement. Additionally, eDNA metabarcoding has also been used for sediment analysis such as the study from Yoccoz et al. [17] where after metabarcoding, terrestrial sediment samples were shown to correctly detect species present at the surface. Moreover, Parducci et al. [29] used metabarcoding on lake sediment cores to detect the ancient plant communities that once lived in the area.

Metabarcoding of air samples has focused primarily on pollen, human health, and forensic geolocation. For example, Korpelainen and Pietilainen [30] used metabarcoding to study the biodiversity of indoor pollen, how it changed over time, and its potential impact on human health. In a comparison of methods, Kraaijeveld et al. [21] found metabarcoding to be more effective at identifying mixed pollen grains than traditional microscopy. Leontidou et al. [31] developed protocols for processing and identifying pollen samples with metabarcoding. Banchi et al. [32] used metabarcoding to simultaneously assess primarily airborne pollen and fungal seasonal diversity across Italy and found that metabarcoding offered promising results for biomonitoring and air quality assessment. More recently, Lennartz et al. [33] examined the plant DNA in dust samples for assisting with forensic geolocation and found that the plant eDNA in settled dust helped provide a reasonable estimate of source location in the United States. These previous works provide a foundation for airborne eDNA monitoring; however, with the understanding that airborne eDNA contains DNA from more than just pollen [22], an eDNA metabarcoding approach offers an opportunity to simultaneously study an entire plant community rather than limiting analysis solely to pollen (apart from Lennartz et al. [33]). If airborne eDNA can be used as an effective whole plant community monitoring method, it could revolutionize the way plant communities are surveyed, invasive species are detected, and endangered species are monitored. Airborne eDNA metabarcoding may be able to detect species more efficiently, with less disturbance, and less effort than traditional surveying. A metabarcoding analysis would also allow us to examine the ecology (origin, state, transport, and fate) of airborne eDNA and how signals change over time to improve development of airborne eDNA methodology [34]. While airborne eDNA and metabarcoding have been used (pollen detection, human health, etc.), there have not been any studies that have examined the ability of airborne eDNA to act as a whole plant community surveying method. Additionally, no studies have compared the results of both an airborne eDNA and traditional whole plant community survey.

Therefore, we provide the first comparison of airborne eDNA metabarcoding and traditional plant survey methods for whole plant community surveying. To understand how airborne eDNA metabarcoding could be useful for plant community surveys, we compared airborne eDNA and traditional plant community surveys over the course of a year to capture the growing and flowering seasons of the species on our study site. The goal of this research was to compare metabarcoding of airborne eDNA to traditional plant surveying in terms of species diversity and effort. Specifically, we wanted to (1) understand which method (metabarcoding or traditional) detected the most species, required the most sampling effort, and what types of species were found; and (2) examine spatial and temporal patterns in airborne eDNA signals over the course of our yearlong survey.

## Results

### Traditional survey

**Fig. 1:**
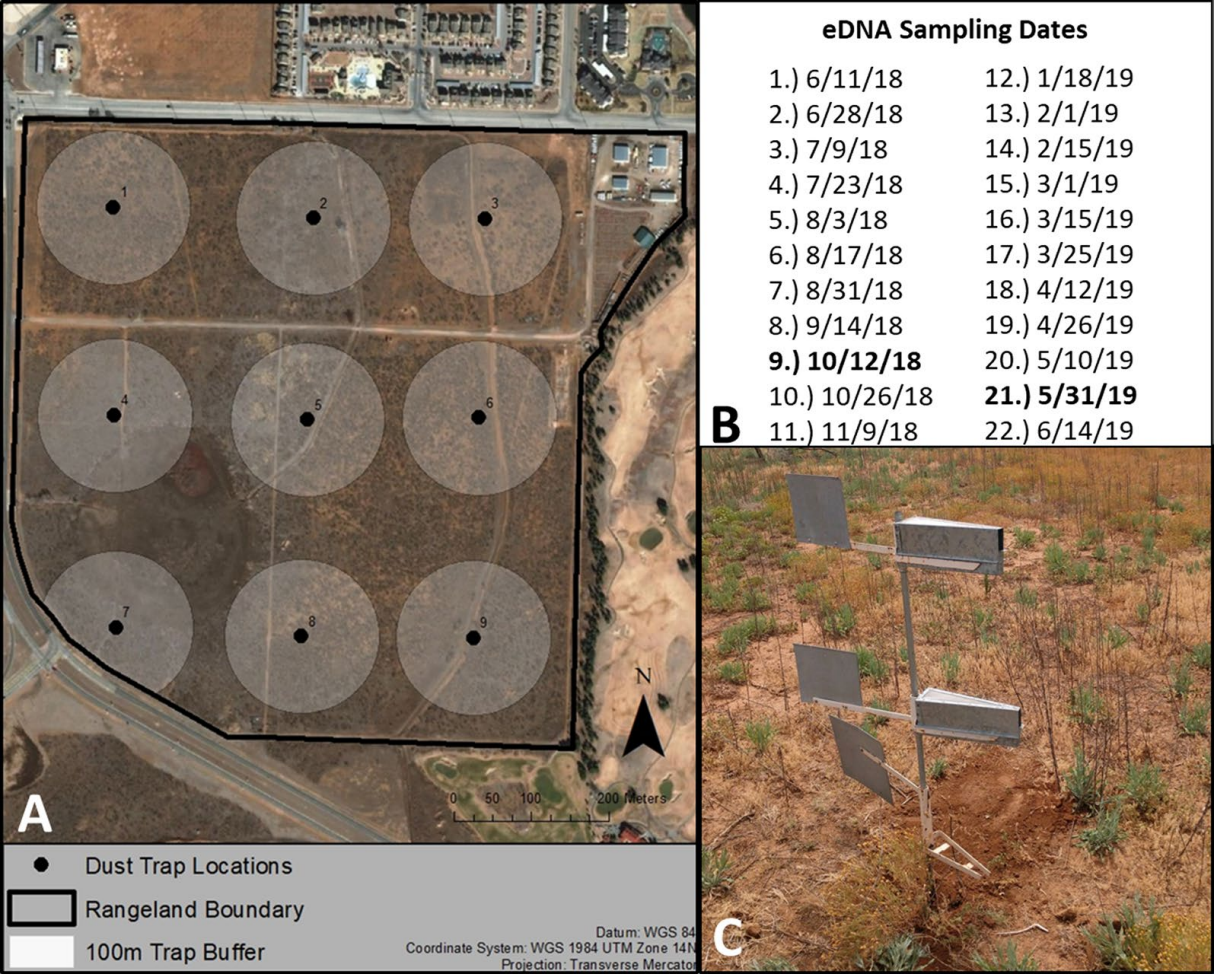
The Texas Tech University Native Rangeland (Lubbock County, TX, United States) study site where both the traditional and metabarcoding plant community surveys were conducted. **A** The points and numbers represent individual Big Spring Number Eight dust trap locations while the buffers around each point represent the 100 m traditional survey extent. For our study site, the wind predominantly blows from the west/northwest to the east/southeast. **B** The sampling dates for our airborne eDNA metabarcoding survey. The dates in bold represent the corresponding times of our two traditional surveys. **C** The Big Spring Number Eight Dust traps that collected the airborne eDNA

**Fig. 2:**
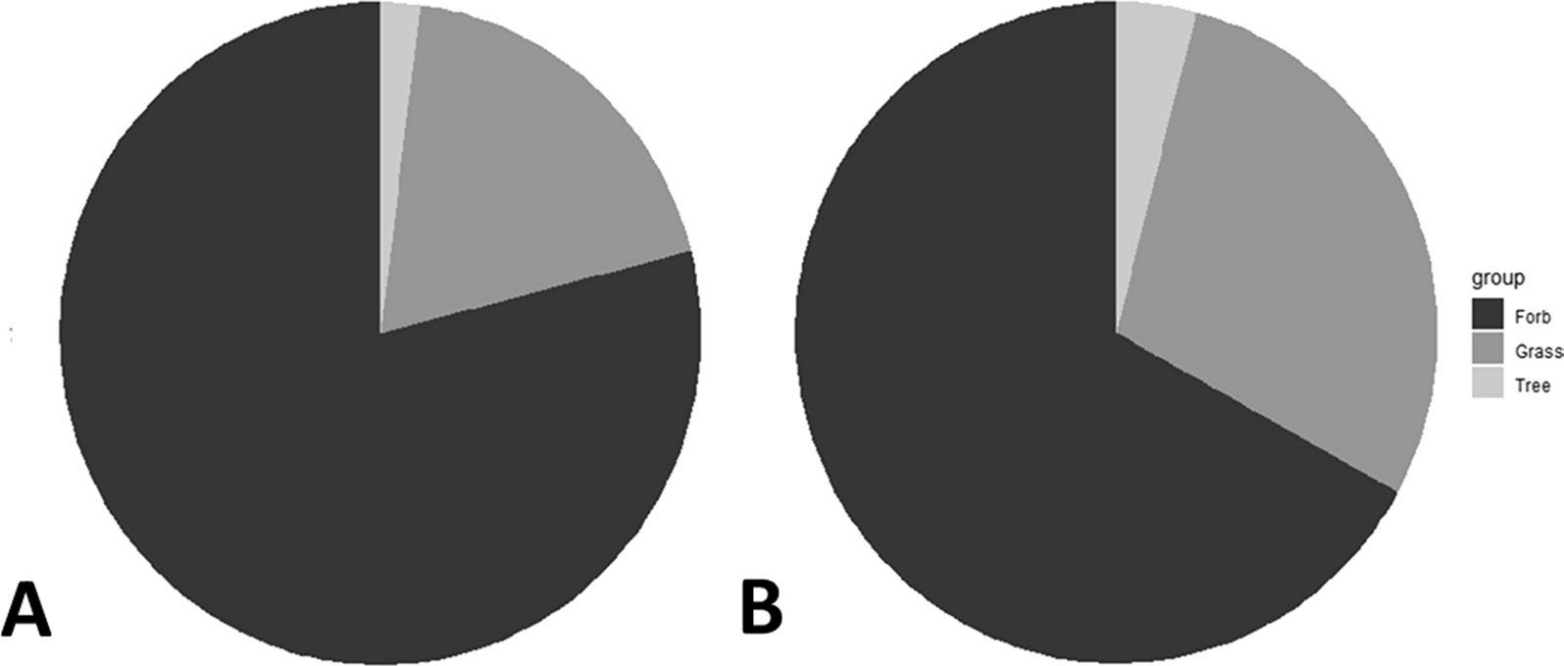
**A** The percentage of forbs (79%), grasses (19%), and trees (2%) that were found during the two traditional surveys on our study site. **B** The percentage of forbs (67%), forbs (29%), and trees (4%) found during the eDNA metabarcoding survey

Over the course of a year, we conducted two traditional transect-based visual plant surveys (September 2018 and May 2019) at the Texas Tech University Native Rangeland consisting primarily of short-grass rangeland habitat (Fig. 1a). Survey methods included a transect-based, line-point intercept survey and broader visual survey methods [2, 3]. The traditional plant community survey conducted in September found a total of 56 species, and the May survey identified 93 species. Overall, when both the September and May surveys were combined, a total of 102 unique species were detected (Additional file 1: Appendix 1). There were 23 specimens from the two surveys unidentifiable due to the plant species being too young or damaged beyond recognition. Furthermore, 22 species lacked reference sequences on NCBI GenBank so they were excluded, leaving 80 total unique species for the methods comparison. In other words, we had no way of knowing if those species were detected with eDNA or not since there was no reference to compare our raw data to. Of the 80 species that were found with the traditional methods, we found 63 forbs (79%), 15 grasses (19%), and 2 trees (2%; Fig. 2a). Across both traditional survey events, the three most common species were *Bouteloua gracilis*, *Prosopis glandulosa*, and *Laennecia coulteri*. For the September survey, the three most common species were *Bouteloua gracilis, Laennecia coulteri*, and *Prosopis glandulosa*, and the three most common species for the May survey were *Bouteloua gracilis*, *Prosopis glandulosa*, and *Salsola tragus* (Table 1). Of the most commonly detected species in the traditional surveys, *Erigeron modestus, Solanum elaeagnifolium, Quincula lobata, and Ammoselinum popei* were not able to be included in the reference library for the metabarcoding survey as there were no *ITS2* sequences available.

**Table 1:** The most common species found in traditional surveys and for each individual survey separately

Traditional survey most common species		
Total	September	May
*Bouteloua gracilis*	*Bouteloua gracilis*	*Bouteloua gracilis*
*Prosopis glandulosa*	*Prosopis glandulosa*	*Laennecia coulteri*
*Laennecia coulteri*	*Salsola tragus*	*Prosopis glandulosa*
*Helianthus ciliaris*	*Helianthus ciliaris*	*Helenium amarum*
*Helenium amarum*	Solanum elaeagnifolium*	Erigeron modestus*
Erigeron modestus*	***Euphorbia lata***	*Lepidium densiflorum*
*Lepidium densiflorum*	*Kochia scoparia*	*Helianthus ciliaris*
*Salsola tragus*	***Hopia obtusa***	*Teucrium laciniatum*
*Solanum elaeagnifolium*	Quincula lobata*	Ammoselinum popei*
*Teucrium laciniatum*	***Portulaca oleracea***	*Plectocephalus americanus*

Sequence data for the species marked with an * were unavailable and thus excluded from comparison with metabarcoding. Additionally, species in bold were not detected by the eDNA surveying method for that respective survey

### Environmental DNA Metabarcoding Survey

We deployed Big Spring Number Eight (BSNE) dust traps at the center of each traditional plant survey location (N = 9; Fig. 1a) to collect airborne eDNA for comparison with traditional survey results. Since airborne eDNA collection is less labor intensive than traditional surveys, we recovered eDNA from traps approximately every two weeks for the same year represented in our traditional plant surveys (i.e., traps were deployed from June 11th 2018 until June 14th 2019, N = 22; Fig. 1b). Across all samples and PCR plates, no contamination was detected in non-template controls and extraction blanks. To ensure that the maximum number of species were detected for our eDNA survey, we performed both a reference library analysis and a BLASTn analysis to expand our taxonomic assignment beyond the list of known plant species at the study site used to create the reference library. The reference library method was able to detect 81 species while the BLASTn analysis found an additional 10 species. Overall, between both the reference library and BLASTn survey, a total of unique 91 species were detected (Additional file 1: Appendix 1). Of these 91 species, we found 61 forbs (67%), 26 grasses (29%), and 4 trees (4%; Fig. 2b).

**Fig. 3:**
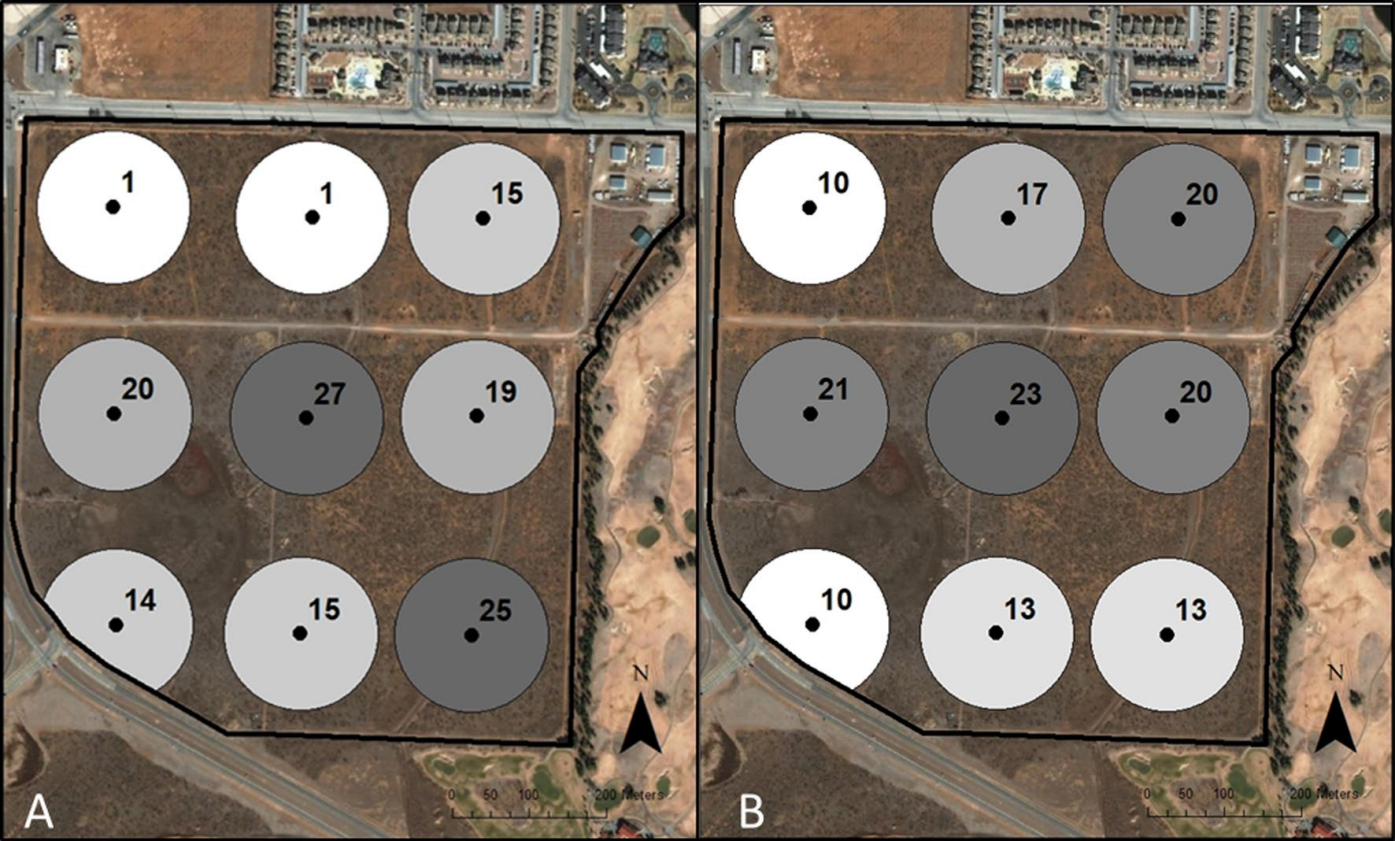
**A** The number of species that each Big Spring Number Eight Dust Traps airborne eDNA trap collected from Event 9 that corresponded to the September survey. **B** The number of species that were captured by each airborne eDNA BNSE trap from Event 21 corresponding to the May traditional survey. The shading represents the amount of species found by each Big Spring Number Eight trap, with the darker coloring indicating more species detected

**Fig. 4:**
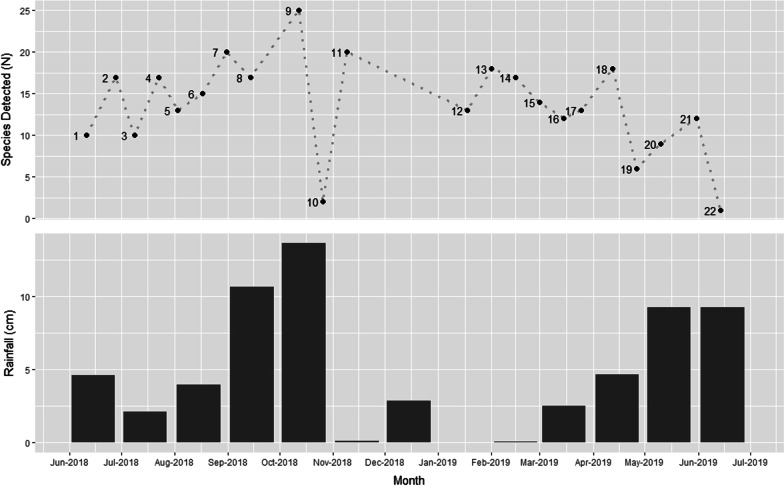
The number of species that were found from event 1 to event 22 during the yearlong airborne eDNA sampling displayed at the top and the amount of monthly rain in centimeters during the same time periods at the bottom

A reference library-based approach relates sequences to a curated database that focuses on a specific DNA region (in our case *ITS2*). Thus, by curating a database of all species known to occur historically on our study site, we could compare the sensitivity of eDNA-based and traditional survey approaches for the detection of a known species. The airborne eDNA samples from each of the nine BSNE traps for each of the 22 sampling events were combined to create 22 combined samples allowing us to examine the overall survey results across the entire year. Additionally, the airborne eDNA from the nine BSNE traps belonging to the two sampling events that corresponded  most closely to the traditional survey were also kept separate. Thus, we could examine the specific trends for each BSNE dust trap from Event 9 (eDNA sampling event corresponding to May survey) and Event 21 (eDNA sampling event corresponded to September survey). We found that the Event 9 samples produced a total of 14,517 reads, detected 39 species, and the three most common species were *Bouteloua gracilis*, *Salsola tragus*, and *Kochia scoparia* (Table 2). The Event 21 samples on the other hand had 63,114 reads, detected 47 species, and found that *Prosopis glandulosa*, *Salsola tragus*, and *Descurainia pinnata* were the top three species detected (Table 2). For the entire eDNA survey, across all sampling events, the reference library-based approach recorded a total of 127,761 reads, 81 species, and the top three species were *Prosopis glandulosa*, *Salsola tragus*, and *Descurainia pinnata* (Table 2). For our study site, the wind predominantly blows from west and northwest down to the east and southeast. We found that both Event 9 and Event 21 shared similar geographical patterns with the lowest species diversity being detected in the northwesterly traps while the largest number of species were found within the center of the rangeland (Fig. 3). Additionally, we tracked the total number of species detected using all eDNA samples (N = 22) across the entire year to determine patterns and trends in the number of species detected (Fig. 4).

**Table 2:** The most common species found for the eDNA metabarcoding reference library analysis in total for Events 9 and 21 separately, which correspond closest to the September and May traditional surveys respectively

Reference library metabarcoding most common species		
Total	Event 9	Event 21
*Prosopis glandulosa*	*Bouteloua gracilis*	*Prosopis glandulosa*
*Salsola tragus*	*Salsola tragus*	*Salsola tragus*
*Descurainia pinnata*	*Kochia scoparia*	*Descurainia pinnata*
*Bouteloua gracilis*	*Cynodon dactylon*	*Helenium amarum*
*Helenium amarum*	*Gutierrezia sarothrae*	*Laennecia coulteri*
***Ulmus pumila***	*Ambrosia psilostachya*	*Machaeranthera tanacetifolia*
*Kochia scoparia*	*Sporobolus cryptandrus*	***Aphanostephus ramosissimus***
*Laennecia coulteri*	***Sorghum halepense***	*Oxalis dillenii*
*Cynodon dactylon*	*Laennecia coulteri*	*Cynodon dactylon*
*Machaeranthera tanacetifolia*	***Verbesina encelioides***	*Ratibida columnifera*

Species in bold represent species that were not found with the traditional surveying method for that respective survey

To examine if we captured airborne eDNA of any plant species not represented within our reference database, a BLASTn survey was also completed. The BLASTn analysis was done with a stricter 100% similarity match to ensure that any species detected were most likely present since they would not be on our reference list. From a total of 144,155 reads, our DADA2 analysis identified a total of 649 amplicon sequence variants (ASVs) which were then examined with BLASTn. Despite having 649 ASVs, only 178 were able to match 100% to a species or genus. From these 178 successfully matched ASVs, a total of 73 species or genera were identified. For our comparison the ASVs that were only identified to genus level were removed from the comparison total values, since no single species could be determined. Of the remaining species, ten were unique species that were not found within our reference library analysis. While our study site is isolated within the city of Lubbock, there are several agriculture fields near our site, a golf course, and several residential living areas that contributed airborne eDNA. For example, the BLASTn survey identified several agricultural crops that exist within a mile of our study site such as cotton (*Gossypium hirsutum*) and soybean (*Glycine max)*. Additionally, the BLASTn survey identified several genera and species of trees that are planted throughout the golf course and residential areas surrounding our study site. This includes the *Pinus* (Pine trees) genus, *Platanus* (Sycamore) genus, cottonwood (*Populus deltoids*), and several oak species (*Quercus* sp.). While these detections are real signals of plant species that exist in the surrounding area, they were excluded in our comparison with the traditional plant survey of the study site.

### Comparison

**Fig. 5:**
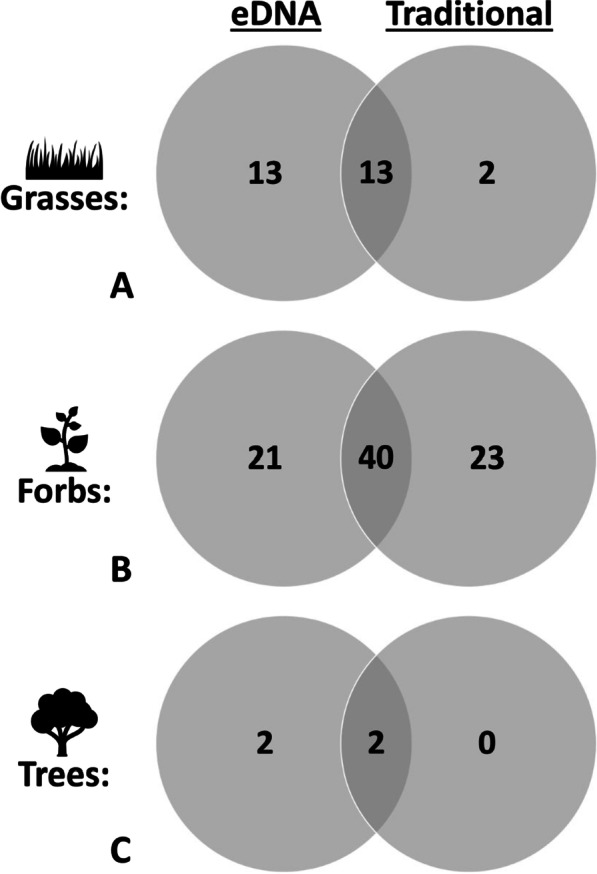
Venn diagram displaying the number of **A** grasses, **B** forbs, **C** and trees that were found by the eDNA and traditional methods alone and together

Overall, our traditional survey found a total of 80 species while the eDNA metabarcoding surveying found 91 species. The types of species (grasses, forbs, trees) that each survey detected were found to vary based on the method being used (Fig. 5). The eDNA metabarcoding survey and traditional survey shared the identification of 13 grasses while the eDNA survey solely detected an additional 13 species compared to just 2 grass species solely detected with the traditional survey (Fig. 5a). Both surveys combined to find 40 shared forbs while our eDNA survey detected 21 unique forbs compared to the 23 detected solely by the traditional survey (Fig. 5b). Lastly, both methods found the same 2 trees while the eDNA survey detected an additional 2 unique species (Fig. 5c).

**Fig. 6:**
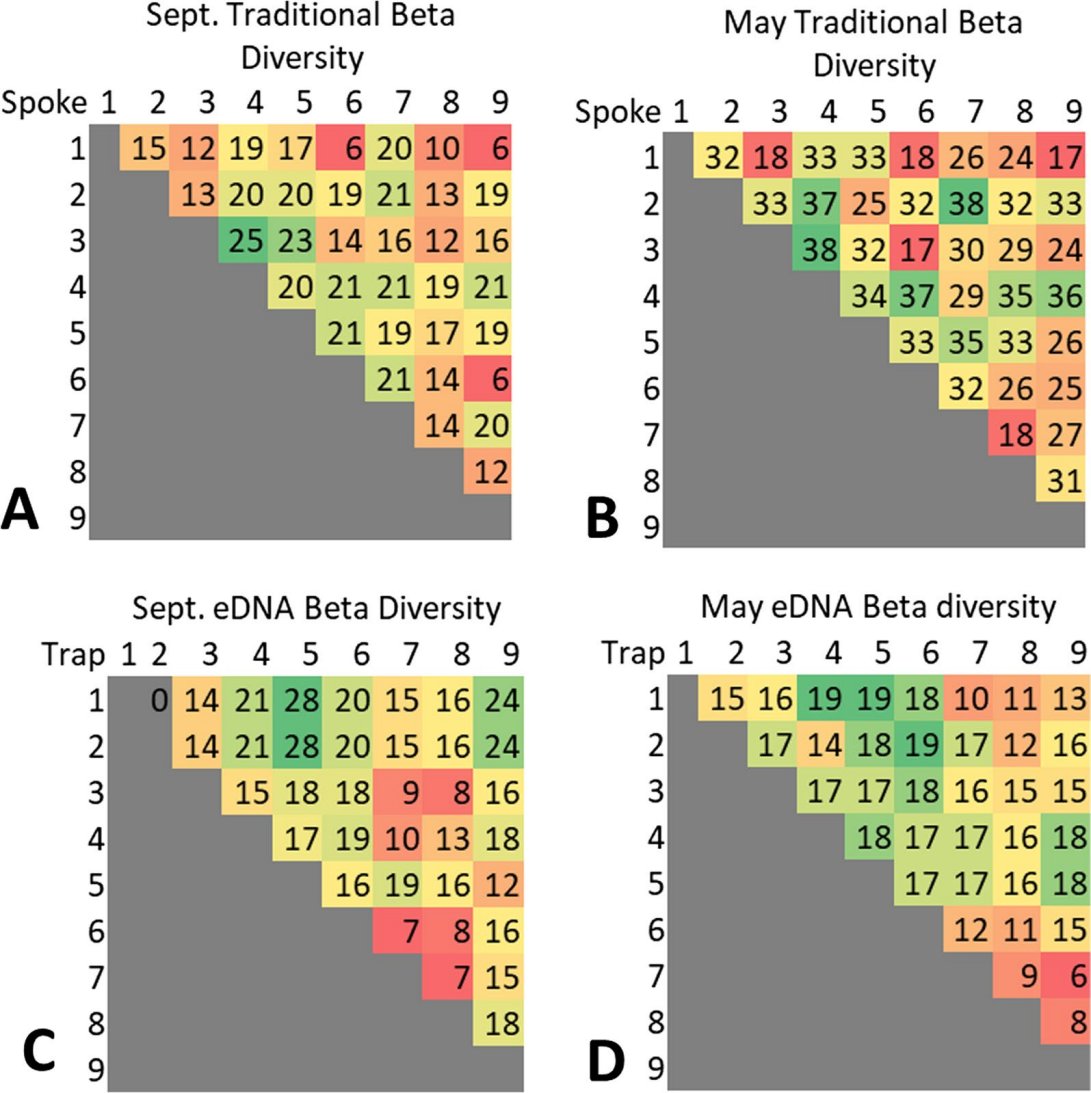
The beta diversity for the **A** September traditional survey, **B** May traditional survey, **C** eDNA sampling Event 9 which corresponds to the September traditional survey, and **D** the eDNA sampling Event 22 which corresponds to the May traditional survey. The coloring for the first column in **C** was removed because the zero represents an outlier

We can also examine the alpha, beta, and gamma species diversity detected by both methods. We compared the alpha, beta and gamma species diversity for the two traditional surveys that took place in September and May and the eDNA sampling Events 9 (September) and 21 (May) only. The alpha diversity is represented by the number of species found within each traditional survey spoke and eDNA trap (Table 3). Furthermore, we can examine the beta diversity between the individual spokes and traps for each survey. The beta diversity in this instance refers to pair-wise comparisons showing the change in diversity between two spokes or two eDNA samplers (Fig. 6). Lastly, the gamma diversity (all unique species across each survey) was described above with the September and May traditional surveys having a gamma diversity of 56 and 93 species respectively. The gamma diversity for the eDNA sampling events 9 and 21 were 39 and 47 respectively.

**Table 3:** The alpha diversity for both traditional surveys (September and May) and the event 9 (September) and event 21 (May) eDNA surveying events

Alpha diversity				
Site/spoke number	Sep. Trad. survey	May Trad. survey	Sep. eDNA event	May eDNA event
1	24	32	1	10
2	31	46	1	17
3	21	31	15	20
4	35	51	20	21
5	37	43	27	23
6	20	30	19	20
7	31	42	14	10
8	29	42	15	13
9	23	33	24	13

## Discussion

Our goal was to determine how an airborne eDNA metabarcoding survey performed compared to a traditional plant community survey. We’ve demonstrated that the airborne eDNA survey was able to detect more species than the traditional survey. We found that both surveys performed differently, finding a different number of species and different types of species. Additionally, we gained knowledge about the ecology of airborne eDNA and how natural factors, such as wind, impacted our ability to collect airborne eDNA.

### Traditional survey compared to eDNA survey

We found that the airborne eDNA survey (reference library and BLASTn) was able to detect more species than the traditional surveying methods, detecting a total of 91 species compared to the 80 species found by the traditional survey. While the airborne eDNA metabarcoding survey found more species, the two surveys varied in the species found, with each method identifying species that were missed by the other and differing greatly in the types of plants detected (Fig. 5). The airborne eDNA survey found 13 grasses not seen in the traditional surveying methods compared to only 2 grasses found with traditional methods not seen in the eDNA survey (Fig. 5a). Grasses predominantly utilize wind pollination, which would release a lot of genetic material and explain the relatively high performance of the eDNA method compared to a traditional survey. Additionally, grasses can be particularly challenging to detect with traditional surveying methods [21], which further explains why eDNA methods would outperform traditional survey methods for this group of plants.

More surprisingly, the eDNA survey detected 21 forbs not seen in the traditional survey compared to 23 forbs detected in the traditional survey not seen in the eDNA survey (Fig. 5b). In general, the eDNA survey tended to detect smaller flowers with a variety of pollination syndromes such as *Ambrosia confertiflora* and *Brickellia eupatorioides*. The forbs that were only detected by the traditional survey were typically rarer plants with large showy flowers such as *Berlandiera lyrata*. This makes sense as the large showy flowers would grab the attention of surveyors during a traditional survey, but if the species is rare, it may not contribute much eDNA to collected samples. Additionally, while both surveys detected cactus DNA, it appears that the traditional survey was better at detecting higher cacti diversity and species that grow slowly such as *Yucca glauca*. It is meaningful to note that while we have identified these trends, some exceptions exist. For example, the eDNA survey detected the larger, showy thistle *Centaurea melitensis*, while the traditional survey detected *Tragopogon dubius*, which is a wind pollinated species. Additionally, the eDNA survey detected more trees than the traditional method, which missed some tree species that were out of eyesight, and the BLASTn results detected multiple tree species’ DNA for species close to our study site (Fig. 5c). This indicates that airborne eDNA could be ideal for tree identification on larger scales as well. Understanding why eDNA and traditional surveys detect different species has been a cornerstone of eDNA research [34], and this is a question that also must be addressed within the airborne eDNA community.

Our results suggest that both traditional and eDNA survey results are influenced by temporal effects such as species seasonality. For example, from the reference library comparison data (Tables 1 and 2), the traditional and eDNA survey share five of the ten most common species across the entire surveys while the September and May surveys each share just three species. Some of these differences can be attributed to the difference in sampling density, which is the result of implementation difficulty. Each traditional survey in May and September required hundreds of volunteer hours and individuals with taxonomic expertise to identify plant species. However, the airborne eDNA survey required about three hours for a single person to collect and filter the airborne eDNA from all nine BSNE traps approximately every two weeks (approximately 66 h for the entire year). Since we were able to sample relatively continuously throughout the year with eDNA, we detected trends in the plant community not observable by our two traditional survey snapshots. In other words, the seasonality and growth patterns of species out on our study site could be tracked much more efficiently with the eDNA method. For example, the eDNA survey found that tansy mustard (*Descurainia pinnata*) was one of the most common species but was rarely seen in the traditional survey (Tables 1; 2). This can be attributed to a large growth and bloom event that was opportunistically observed during the eDNA surveying early in the spring, but not captured by either formal traditional survey. For a month, our study site was dominated by this species, but by the time the traditional survey took place in May there was little remaining to indicate this. This highlights how the nature of an airborne eDNA survey being easier to implement and sample allowed for more species to be detected on our study site.

In addition to detecting more species, the eDNA survey also detected more invasive species than the traditional survey. On our study site there are three major invasive species, Russian thistle (*Salsola tragus*), Mexican feather grass (*Nassella tenuissima*), and tree of heaven (*Ailanthus altissima*). While both the traditional and eDNA surveys found Russian thistle and Mexican feather grass, only the eDNA survey detected tree of heaven. This is meaningful because tree of heaven represents an invasive species that is in the ideal stage for eradication. The most effective time to stop an invasive species is before it becomes established, which can be challenging if traditional methods are unable to detect an invasive fast and efficiently enough [35]. In our study, airborne eDNA was able to detect the tree of heaven when it was rare and not established while the traditional survey did not. In addition to showing that airborne eDNA may be ideal for the detection of invasive species early in the establishment process, airborne eDNA may also help detect endangered species that are also rare in the environment.

Overall, the airborne eDNA survey was able to detect more species than the traditional survey, required less sampling effort, and detected more invasive species. However, it has been shown that oftentimes the best way to utilize eDNA surveying is in conjunction with traditional methods due to the strengths and weaknesses of each method [9, 36]. For example, 22 species were not included in the traditional surveying method due to no reference data existing for them. While these were removed from the comparison because there is no way to know if we detected those species or not, these still constitute real species detected by the traditional survey. As time goes on the number of species without reference information will continue to drop, but for the time being the restriction of sequencing data is a limitation of eDNA surveying. By combining both methods, a more well-rounded surveying approach can be established.

### Other patterns

In addition to examining the number and types of species our surveys detected, this study has also shed light on the ecology of airborne eDNA, which refers to the origin, state, transport, and fate of eDNA in the environment [34]. One of the most insightful aspects of our study for the understanding of airborne eDNA analysis is that we were able to sample repeatedly over the course of an entire year, which had never been done at this temporal scale before. As a result, we observed how species signals changed throughout the year. For example, blue grama (*Bouteloua gracilis*) was the most detected species in Event 9, the fourth most detected species overall, and not in the top ten for Event 21 (Table 2). However, this species was the most common for the total survey and both sub-surveys for the traditional survey (Table 1). This can be explained by examining the ecology of blue grama, which is pollinated in the fall (Event 9) and then goes dormant in the early summer (Event 21). During this dormancy, it is not growing, flowering, or pollinating, which minimizes its production of airborne eDNA [23].

Another example of the ecology of a species impacting detection is Russian thistle (*Salsola tragus*). Russian thistle was the second most common species detected with our eDNA survey across both sampling events and the total survey (Table 2). However, this species was not within the top ten most commonly detected species in the May traditional survey and was only common in the September traditional survey (Table 1). These patterns can be explained by examining the unique lifestyle of Russian thistle. Russian thistle grows in the spring where it flowers and forms seeds. Once the seeds are formed, the plant breaks off the ground and tumbles across the landscape spreading up to 50,000 seeds and oftentimes getting caught on fence lines [37]. With the plant having various life stages and moving positions, it is hard for the traditional survey to consistently find the species. However, since the species is moving so much and releasing so much material, the eDNA metabarcoding survey was able to consistently detect this species.

We also found that the time of year greatly impacted the number of reads sequenced. For example, our samples from Event 9 in the fall produced 14,517 reads for reference library analysis while Event 21 in the spring produced 63,114 reads. This is most likely because in spring more species are growing, flowering, and releasing large plumes of eDNA while in the fall, most species are done flowering and preparing to die or experience dormancy over the winter months. Furthermore, the trend of springtime producing more data is shown with Event 21 detecting seven more species than were found with Event 9. We can also examine the number of species that were found at each combined collection time across the entire year (Fig. 4). The number of species found on average with each event was approximately 14 and stayed between 10 and 20 species for most of the events sampled. The large dip on October 26, April 26, and May 10 can be explained by large rain events occurring near or during the time of sampling. Johnson et al. [22] found that rain appeared to have an impact on the amount of airborne eDNA that could be collected which is also shown in our results. The lowest dip on June 14 however was not caused by rain and may be the result of data being lost during the combination of the nine BSNE traps, a dilution error, or user error during the metabarcoding pipeline. Lastly, we can see that combining the nine BSNE traps into a single combined sample led to less species detected per event. The nine BSNE dust traps were analyzed separately for events 9 and 22 and found 39 and 47 species, respectively with a total of 65 unique species when combined. This was much higher than any of the species number we detected with combined samples (Fig. 4). While combining the samples allowed us to survey over a longer period and detect species throughout the year (and detect more species than the traditional survey), we would recommend that future projects use as many samples as possible and avoid pooling samples if the funds are available.

In addition to examining how reads and the number of species changed temporally, our separate reference library analysis of Event 9 and Event 21 allows us to examine how each trap performed spatially. Across both events, the general trend was that the more northwesterly trap detected the fewest number of species, which can be explained by the fact that the wind blows in from the northwest, and any material that is carried on the wind in that corner of the study site will not be from our rangeland (Fig. 3a and b). The traps in the center of the rangeland for both events collected the most species, and the number of species typically increased toward the east in line with the wind. This highlights that airborne eDNA collection must consider local factors that can quickly change such as the weather.

We also saw interesting trends when it came to the spatial distribution of our eDNA detections through the beta diversity analysis (Fig. 6). A spoke or eDNA trap with a consistently higher beta diversity indicates that the location is detecting more unique species then other spokes or traps. We found that spokes 4 and 7 both had consistently high beta diversity numbers. This trend can be attributed to the direct environment in which these transects were located. For example, both transects 4 and 7 are adjacent to the playa lake located on the property and detected multiple species unique to the playa and the land around it (Fig. 1). On the other hand, spokes 3, 6 and 9 typically had low beta diversity outputs because these environments had little diversity as shown with their alpha diversity (Table 3). The eDNA beta diversity results on the other hand found a different trend, with traps 5, 6, and 9 having high beta diversity metrics which is in direct contrast to the traditional survey results. This increase in beta diversity may be attributed to the idea that eDNA is able to integrate the landscape’s eDNA signals within an area that eDNA pools. For example, Deiner et al. [38] found that aquatic eDNA can be detected in the catchments of rivers, with the rivers acting as conveyor belts. In our case, the wind is our conveyor belt, with traps like 5, 6, and 9 all being directly in the south-east direction of the wind as it travels over our study site. By collecting airborne eDNA from the traps that are at the end of the wind flow, we can maximize the number of unique species that the wind picked up as it moved across our study site.

Additionally, throughout the year there were multiple species that were consistently detected regardless of their pollination syndrome (wind or insect pollinated), flowering season, or time of year. Some examples include honey mesquite (*Prosopis glandulosa),* Coulter’s horseweed (*Laennecia coulteri)*, Russian thistle, and Johnson grass (*Sorghum halepense)* which were found throughout the combined yearlong samples. Furthermore, multiple species were detected that are primarily insect pollinated such as tansy mustard and honey mesquite which were two of the three most common species in our reference library analysis (Table 2). This confirms previous findings that airborne eDNA can be used to detect species from more than pollen in the air and is most likely detecting leaf fragments, flower fragments, and free floating DNA alongside pollen [22].

## Conclusions

Overall, compared to a traditional plant community survey approach, the eDNA survey found more species, detected more invasive species, and was able to survey our study site more often due to its relatively low time and resource requirements. Thus, we believe airborne eDNA surveillance represents a valuable contribution to the toolbox for the study and management of terrestrial plant communities. Recently, there has also been evidence supporting the presence of animal eDNA in the air, indicating airborne eDNA could be applied to terrestrial animal surveying [39]. The application of airborne eDNA monitoring could be applied to areas such as invasive and endangered species detection, understanding how a disturbance (fire, storm, flood, etc.) impacts a community, and tracking the change in communities over time due to climate change. In the future more research needs to be done on the ecology of airborne eDNA and expanding this method into other life forms such as animals. As we have shown, airborne eDNA represents an especially useful plant community monitoring tool that should be further explored by researchers and managers.

## Methods

### Study site

Surveys were conducted on the approximately 53-hectare Texas Tech University Native Rangeland (33.60327 N, -101.9003 W). This rangeland, located within the city of Lubbock, Texas, USA, is used and maintained as a research and teaching resource by the Texas Tech Department of Natural Resource Management (Fig. 1a). This site is primarily a short-grass prairie with many native bunch grasses, forbs, and cacti. Additionally, the site has a large population of honey mesquite and several documented invasive species including Russian thistle (*Salsola tragus*), Mexican feather grass (*Nassella tenuissima*), and tree of heaven (*Ailanthus altissima*). In addition to the short-grass prairie habitat, the site also contains an ephemeral playa lake, which hosts several wetland plant species. A benefit of using the Texas Tech University Native Rangeland is that this study area had a list of 165 plant species that had been observed over the course of the last two decades by professors and classes that have taken place on the property.

### Traditional survey

We conducted a traditional plant community survey at two different times of the year to capture both the spring and fall flowering seasons. The first survey occurred from September 21 to 25, 2018, followed by a second survey from May 23 to 24 2019. To capture plant community diversity, cover, and abundance, we established 9 sampling locations, each consisting of three 100 m transects organized as spokes around a central point as recommended by Herrick et al. [3]. The spoke designs help to reduce disturbance along the transects by focusing the disturbance to the center of the site [3]. The location and size of the spokes were chosen to provide the most coverage of the rangeland property while minimizing site overlap [2]. Each sampling location consisted of three equidistant 100 m transects spreading out in three random equidistant directions from a central point. These three transects result in coverage of approximately 3.1 hectares per sampling location, and a total of nine sites were set up across the rangeland in a grid formation (Fig. 1a).

At each transect within each site (n = 27 total transects), two separate plant community survey methods were used: a line-point intercept survey followed by a broader visual survey. The line-point intercept survey uses a pin to measure plant diversity and abundance. Starting at a randomly selected point within the first meter of each transect, a pin was dropped at 1-m intervals, and we recorded all vegetation that intercepted or touched the pin. The line-point intercept method is reliable for determining the species found along a transect. However, the goal of this survey was to find the most species possible, so a visual survey was also conducted along the length of each transect. Any species found near our transects but not captured by the line-point intercept survey were also recorded via this second survey approach.

### Airborne eDNA collection, extraction, amplification, and sequencing

To collect airborne eDNA, we deployed Big Spring Number Eight (BSNE) dust traps at the center of each site (N = 9) for the traditional survey (Fig. 1a; [22–24]). Each BSNE trap consisted of two independent triangular metal collectors approximately 0.9 and 0.4 m above the ground (Fig. 1c). Each collector included a metal sail that aligned the opening at the front of the collector into the wind to collect dust and particles at the bottom of the trap. These traps were the most efficient passive method for collecting airborne eDNA in a previous methods comparison at our study site [23]. We completed the airborne eDNA survey during the same year (2018–2019) as the traditional surveys. The airborne eDNA collection was much less labor intensive than the traditional survey methodology, so we recovered eDNA from traps approximately every 2 weeks for a year. Specifically, the traps were deployed from June 11th 2018 until June 14th 2019, with 22 collection events in between.

To recover the airborne eDNA from a BSNE dust trap, we used approximately 1 l deionized water to wash the material out of the bottom of each collector into a sterile 1-l bottle. Both collectors on each BSNE trap were combined into a single sample, with each trap contributing approximately 500 ml to the overall 1-liter sample. We transported the rinse water to the laboratory in a cooler and vacuum filtered the samples through 1 μm Isopore membrane filters within 2 h of collection. Filters were stored at − 20 °C until the DNA extractions took place. We extracted total genomic DNA using a DNeasy PowerPlant Pro DNA Isolation Kit (QIAGEN), which demonstrated high efficiency in previous airborne eDNA analyses [23]. We followed the manufacturer’s protocol, except we added an extra grinding step with a sterile plastic pestle and frequent vortex agitation to ensure homogenization at the beginning of the extraction process [22–24]. Extracted genomic DNA was stored at − 20 °C until future DNA analysis. To ensure that there was no contamination throughout this process, extraction blanks were used for each extraction event (N = 11) along with sterile gloves, containers, and forceps.

Following DNA extraction, we pooled genomic DNA from all nine BSNE traps combined for each sampling event, creating 22 combined airborne eDNA samples. We also analyzed a subsample from each of the nine BSNE traps from sampling events on October 12, 2018 (“Event 9”) and May 31, 2019 (“Event 21”) individually to examine how the specific eDNA sampling events that were most closely paired in time with traditional plant surveys. Event 9 and Event 21 both correspond to the fall and spring traditional surveys, allowing us to make a more detailed comparison. Thus, 40 samples were analyzed overall: 22 combined samples representing the entire landscape at each airborne eDNA sampling event, and 18 samples representing each individual sampling site on two specific dates. Finally, we diluted samples by a factor of 10 with pure deionized water to limit PCR inhibition, which we have commonly observed in airborne eDNA samples at our study site [22–24].

Samples were amplified with polymerase chain reaction using a QuantStuido 3 Real-Time PCR System (ThermoFisher Scientific). We targeted the nucleic *ITS2* marker because it corresponded to an abundance of available NCBI GenBank sequences relative to other plant barcoding genes (e.g., *trnL*, *rbcl*, *matK*). Specifically, we amplified DNA from our samples using the forward S2F and reverse ITS4 primers as described by Keller al. [40]. Each 50 µl reaction contained 1X AmpliTaq Gold 360 Master Mix (ThermoFisher Scientific), 0.16 µmol/l forward and reverse primer concentrations, and 3 µl template. The thermocycling program began with an initial 95 °C step for 10 min, followed by 40 cycles of 40 s at 95 °C, 40 s at 49 °C, and 40 s at 72 °C. Each plate contained a negative control to ensure no contamination took place during experimental setup. Amplified products were visualized via electrophoresis on a 2% agarose gel stained with GelRed (Bio-Rad). Extraction blanks were examined following the same protocol.

We cleaned and extracted amplified products from the gel with the PureLink Quick Gel Extraction Kit (Thermo Fisher Scientific). Next, we prepared each library for Illumina MiSeq amplification using the QIAseq 1-Step Amplicon library kit (QIAGEN), which ligated unique barcoded adaptors onto the ends of the DNA in each sample, while also removing excess adapters, dimers, and other contaminants. Once library preparation was completed, we quantified samples with a Qubit Fluorometer (ThermoFisher Scientific), and the fragment size for each library was determined using a TapeStation System (Agilent). Libraries were then diluted to 10 nM, and pooled into a single sample for even coverage during sequencing [41]. Finally, paired-end sequencing was carried out with an Illumina MiSeq (Illumina, San Diego, CA, USA). In preparation for bioinformatic analysis, reads were demultiplexed and exported as a FASTQ files.

### Taxonomic identification and comparison

We analyzed sequences by first using a reference library approach then using a BLASTn approach to capture any species not included in our reference library. For the reference library approach, we searched NCBI GenBank for *ITS2* sequences from all plant species known to occur on the Texas Tech University Native Rangeland (N = 165) and downloaded available sequences as FASTA files. Sequences were mapped against the reference database using SeqMan NGen (DNASTAR INC). The software automatically aligned our data, trimmed our reads, and removed low quality redundant sequences. We selected a minimum required base pair assignment of 190 base pairs to limit assignment errors and added a successful match threshold of 99% [42]. In addition to taxonomic identification of each sequence, at the end of the analysis, the number of reads from each sample were recorded for each matched reference library accession number. Additionally, the reference library data for specifically Events 9 and 21 were analyzed alongside the traditional survey data to produce alpha, beta, and gamma diversity metrics.

To identify species using a BLASTn approach, demultiplexed Illumina sequences were processed using DADA2 [43]. First, both the forward and the reverse sequences were filtered and trimmed based on a generated quality profile. Next, the DADA2 algorithm used a parametric error model to determine likely errors in the sequence data and filter out those errors. Paired reads were merged (12 base pair overlap and no mismatches), chimeras were removed, and ASVs were output. The ASVs are an output from DADA2 and considered a higher resolution analogue to operational taxonomic units (OTU) and show the unique sequences found within your samples and the number of reads associated with each ASV [43]. Using ASVs allowed our BLASTn analysis to be completed much more efficiently than if we had examined all of our sequences individually (144,155 reads). Finally, ASVs with 10 sequences or fewer were removed from our future BLASTn analysis to limit sequencing and assignment errors [44]. Taxonomic assignment for the remaining unique ASVs was completed using BLASTn and the NCBI nt database. Because the BLASTn approach is not influenced by a priori observation of species on the landscape like the reference library approach, we required 100% identity match for taxonomic identification. If any ASVs had a 100% match to multiple species in the same genus, the ASV was assigned to the genus level [45]. For ASVs that had multiple 100% genera or species matches, all were kept following the recommendation of Klymus et al. [46]. The species and genus results were then examined through the United States Department of Agriculture Plants Database [47] to classify the species or genus as being either within our study site (i.e., Texas Tech Native Rangeland), local county (Lubbock County), state (Texas), country (United States), or the World. Once labeled, the results were examined and species that were classified as being within our rangeland or Lubbock County along with making ecological sense were considered to be a positive detection from the BLASTn survey. In this instance, the term “ecological sense” involves examining the detected species and determining if it is likely to exist on our rangeland. For example, we had sequences from several agricultural fields, but there is no cotton, for example, actually growing within our study site. The ASVs that could detect genera were examined separately from species assignments. Lastly, the total number of species and the types of species found were compiled for both the traditional and metabarcoding methods and compared.

## Supplementary Information

**Additional file 1:** Appendix.

## Data Availability

Sequence results are added as an Additional file 1: Appendix on this manuscript. Raw FASTQ files are stored with the Texas Tech University Dataverse as part of the Texas Data Repository and can be accessed via https://doi.org/10.18738/T8/FKQVUG.

## Abbreviations

eDNA: Environmental DNA
BSNE: Big Spring Number Eight dust traps
ASV: Amplicon sequencing variant
*ITS2*: Nternal transcribed spacer 2
*trnL*: Hloroplast *trnL* (UAA) intron
